# Relationship between depressive disorders and biochemical indicators in adult men and women

**DOI:** 10.1186/s12888-023-04536-y

**Published:** 2023-01-18

**Authors:** Xinyuan Li, Yafei Mao, Shumin Zhu, Jin Ma, Shichao Gao, Xiuyu Jin, Zishuan Wei, Yulan Geng

**Affiliations:** 1 Department of Laboratory Medicine, The First Hospital of Hebei Medical University, 89 Donggang Road, Shijiazhuang, 050031 China; 2 Department of Laboratory Medicine, The Third Hospital of Hebei Medical University, Shijiazhuang, China; 3 Research Center, The Fourth Hospital of Hebei Medical University, Shijiazhuang, China; 4 Department of Laboratory Medicine, The First Hospital of Hebei Medical University, 89 Donggang Road, Shijiazhuang, 050031 China

**Keywords:** Depressive disorder, Biochemistry profile

## Abstract

**Background:**

Depression is a psychiatric disorder with global public health concerns. Although a number of risk factors have been identified for depression, there is no clear relationship between biochemistry and depression. In this study, we assessed whether depressive disorders are significantly associated with biochemical indicators.

**Methods:**

Our study included 17,561 adults (age ≥ 18 years) participating in the 2009-2018 National Health and Nutrition Examination Survey (NHANES). The relationship between depression and biochemical and obesity indicators was analyzed by logistic regression.

**Results:**

As compared to the control group, men with depression showed significantly higher levels of gamma-glutamyl transferase, glucose, and triglycerides, and lower levels of albumin and total bilirubin. The depressed group had higher levels of alkaline phosphatase, bicarbonate, and sodium than the control group.

**Conclusion:**

Several biochemical and anthropometric indices were associated with depression in this study. It would be interesting to further analyze their cause-effect relationship.

**Limitations:**

This study is a cross-sectional study. The population is less restricted and does not exclude people with diabetes, pregnancy, etc., so it is less significant for a specific population. Dietary information was not included, as diet plays an important role in many indicators.

## Introduction

There is widespread public health concern about depression, especially in developing countries [1]. It is expected that depression will contribute the most to the burden of disease by 2030 [2]. There are several diseases associated with depression, including suicide, obesity, hypertension and stroke, cardiovascular disease, and Alzheimer’s disease [2–4]. The main known risk factors for depression are gender (most common in women), low education level, low income, smoking, nicotine dependence symptoms, alcohol consumption, body mass index (BMI), waist circumference, triglycerides, glucose, total cholesterol, blood urea nitrogen (BUN), genetic factors, etc. Several studies have reported a correlation between gender and body mass index and depression [5, 6]. But the correlation between depression and biochemically related indices is not clear, for example, triglyceride levels are strongly associated with depression [7], but not with depression [8]. Therefore, this study aimed to examine whether depressive disorders are significantly associated with biochemical indicators in men and women separately in a large cross-sectional study and to determine whether multiple biochemical markers can discriminate between depressed patients. It may be possible to gain a better understanding of depressive disorders in the US population based on our findings.

## Methods

### Study population

**Fig. 1:**
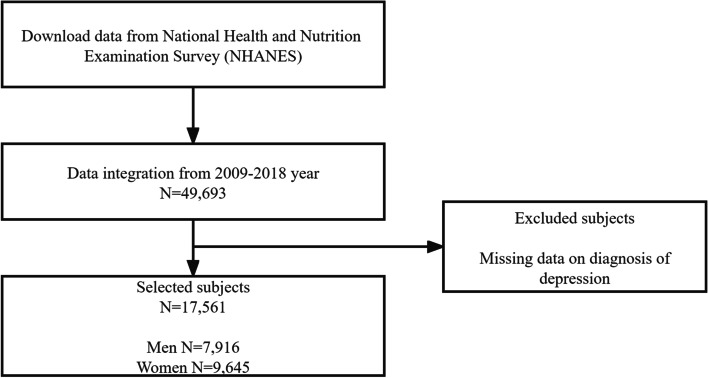
Sample selection procedure

Those participating in the study were from the National Health and Nutrition Examination Survey (NHANES), which aimed to assess the health and nutrition status of adults and children in the United States. In this cross-sectional study, we used data from NHANES 2009-2018. Participants were selected from 49,693 surveyed residents based on selection criteria. Finally, our study analyzed 17,561 participants after the exclusion criteria: 1) lack of information on depression (Fig. 1).

### Measurements

#### Depression

The Patient Health Questionnaire-9 (PHQ-9) was used as an independent variable in this study to measure depression symptoms in the past 2 weeks [9]. The PHQ-9 items range from 0 (not at all) to 3 (almost every day) and the total score ranges from 0 to 27. A meta-analysis found that defining depression based on a score of 10 maximized composite sensitivity and specificity [10]. Additionally, patients with major depressive disorder rarely score below 10 [9]. The participants classified as depressed were those who gained a total score of 10 or above.

#### Covariates

In the demographic questionnaire, age, gender, race, education level, marital status, and family poverty income ratio, body mass index, smoking status, alcohol intake status, whether to drink too much alcohol and waist circumference were included among the demographic characteristics. Based on the questionnaire responses, alcohol intake status was classified as never drinking, former drinking, and whether to drink too much alcohol [11].

### Statistical analysis

All analyses were performed using R 3.3.2(http://www.R-project.org, R base) and Free Statistics version 1.5. Data were compared using the Mann-Whitney test for continuous variables and the chi-square test for categorical variables. The chi-square test was used to compare categorical variables between the control and depressive disorder groups. Binary logistic regression was used for standardized transformed correlations (mean = 0, standard deviation = 1) between the control group and the depressive disorder group with two covariates (age and race) for the crude model. Model 1 included the covariates from the crude model plus income, education, marital status, whether alcohol was used, and female drinking status. Model 2 included the covariates of model 1, plus BMI and waist circumference. Ratio ratios are expressed as 95% confidence intervals (ci), and *p* values < 0.05 were considered significant.

## Results

The sociodemographic characteristics of the study sample are shown in Table 1. Out of 49,693 participants in NHANES 2009-2018, 17,561 were included in the analysis. The study population included 6857 men and 7931 women in the control group, 1059 men and 1714 women in the depression group (Fig. 1). Age, race, income, education, marital status, BMI, waist circumference, and alcohol abuse were significantly associated with depression in men (Table 2). Drinking status was associated with depression significantly more in women than in men, and the rest was associated with depression significantly in men as well (Table 3).

**Table 1:** Gender differences in the full sample

Variables	Total (*n* = 17,561)	Non-depression (*n* = 14,788)	Depression (*n* = 2773)	*p*	Statistic
Gender, n (%)				< 0.001	63.096
Men	7916 (45.1)	6857 (46.4)	1059 (38.2)		
Women	9645 (54.9)	7931 (53.6)	1714 (61.8)

**Table 2:** Sociodemographic characteristics of the male study sample

Variables	Total (*n* = 7916)	1 (*n* = 6857)	2 (*n* = 1059)	*p*	Statistic
Age, Mean ± SD	46.9 ± 18.7	46.5 ± 18.9	49.0 ± 17.7	< 0.001	16.217
Race, n (%)				0.001	17.581
Mexican American	1165 (14.7)	1028 (15)	137 (12.9)		
Other Hispanic	736 (9.3)	616 (9)	120 (11.3)		
Non-Hispanic White	3339 (42.2)	2878 (42)	461 (43.5)		
Non-Hispanic Black	1605 (20.3)	1376 (20.1)	229 (21.6)		
Other Race - Including Multi-Racial	1071 (13.5)	959 (14)	112 (10.6)		
Ratio of family income to poverty, Mean ± SD	2.4 ± 1.6	2.5 ± 1.6	1.8 ± 1.4	< 0.001	182.806
Education level, n (%)				< 0.001	91.744
Less Than 9th Grade	883 (11.6)	722 (10.9)	161 (15.9)		
9-11th Grade (Includes 12th grade with no diploma)	1714 (22.4)	1427 (21.5)	287 (28.4)		
High School Grad/GED or Equivalent	1730 (22.6)	1496 (22.6)	234 (23.1)		
Some College or AA degree	1821 (23.8)	1594 (24)	227 (22.5)		
Some College or AA degree	1491 (19.5)	1389 (21)	102 (10.1)		
Marital status, n (%)				< 0.001	86.871
Married	4472 (57.6)	3999 (59.5)	473 (45.6)		
Widowed	640 (8.2)	518 (7.7)	122 (11.8)		
Divorced	885 (11.4)	713 (10.6)	172 (16.6)		
Separated	204 (2.6)	164 (2.4)	40 (3.9)		
Never married	987 (12.7)	834 (12.4)	153 (14.7)		
Living with partner	575 (7.4)	497 (7.4)	78 (7.5)		
Body Mass Index, Mean ± SD	28.9 ± 6.5	28.8 ± 6.4	29.6 ± 7.2	< 0.001	12.761
Waist Circumference..cm., Mean ± SD	101.2 ± 17.0	100.8 ± 16.8	103.5 ± 18.4	< 0.001	21.467
Alcohol intake status, n (%)				0.679	0.775
Current drinking	1090 (45.5)	929 (45.1)	161 (47.4)		
Former drinking	791 (33.0)	680 (33)	111 (32.6)		
Never drinking	517 (21.6)	449 (21.8)	68 (20)		
Whether to drink too much alcohol, n (%)				< 0.001	11.979
Moderate current drinking	5254 (91.3)	4620 (91.8)	634 (87.9)		
Excessive current drinking	499 (8.7)	412 (8.2)	87 (12.1)		
Smoking status, n (%)				0.245	2.813
Current smoking	975 (21.3)	836 (21.2)	139 (21.9)		
Former smoking	1087 (23.8)	922 (23.4)	165 (26)		
Never smoking	2512 (54.9)	2182 (55.4)	330 (52.1)

**Table 3:** Sociodemographic characteristics of the female study sample

Variables	Total (*n* = 9645)	1 (*n* = 7931)	2 (*n* = 1714)	*p*	Statistic
Age, Mean ± SD	47.7 ± 18.6	47.5 ± 18.8	48.5 ± 17.4	0.05	3.826
Race, n (%)				< 0.001	24.1
Mexican American	1397 (14.5)	1122 (14.1)	275 (16)		
Other Hispanic	1111 (11.5)	881 (11.1)	230 (13.4)		
Non-Hispanic White	3907 (40.5)	3230 (40.7)	677 (39.5)		
Non-Hispanic Black	2079 (21.6)	1704 (21.5)	375 (21.9)		
Other Race - Including Multi-Racial	1151 (11.9)	994 (12.5)	157 (9.2)		
Ratio of family income to poverty, Mean ± SD	2.3 ± 1.6	2.4 ± 1.6	1.7 ± 1.4	< 0.001	281.981
Pregnancy status at exam, n (%)				0.224	1.479
Yes	215 (5.7)	186 (5.9)	29 (4.7)		
NO	3542 (94.3)	2952 (94.1)	590 (95.3)		
Education level, n (%)				< 0.001	111.894
Less Than 9th Grade	1020 (11.0)	768 (10)	252 (15.2)		
9-11th Grade (Includes 12th grade with no diploma)	2202 (23.7)	1745 (22.8)	457 (27.5)		
High School Grad/GED or Equivalent	2080 (22.3)	1721 (22.5)	359 (21.6)		
Some College or AA degree	2296 (24.7)	1881 (24.6)	415 (25)		
Some College or AA degree	1712 (18.4)	1535 (20.1)	177 (10.7)		
Marital status, n (%)				< 0.001	121.746
Married	4762 (50.2)	4105 (52.6)	657 (39)		
Widowed	1330 (14.0)	1051 (13.5)	279 (16.5)		
Divorced	1289 (13.6)	1002 (12.8)	287 (17)		
Separated	327 (3.4)	231 (3)	96 (5.7)		
Never married	1149 (12.1)	925 (11.9)	224 (13.3)		
Living with partner	628 (6.6)	485 (6.2)	143 (8.5)		
Body Mass Index, Mean ± SD	30.2 ± 8.2	29.8 ± 7.9	31.9 ± 9.0	< 0.001	94.711
Waist Circumference..cm., Mean ± SD	98.6 ± 17.6	97.8 ± 17.3	102.3 ± 18.5	< 0.001	89.081
Alcohol intake status, n (%)				0.018	8.016
Current drinking	1255 (27.4)	1056 (27.8)	199 (25.3)		
Former drinking	1759 (38.4)	1422 (37.4)	337 (42.8)		
Never drinking	1572 (34.3)	1321 (34.8)	251 (31.9)		
Whether to drink too much alcohol, n (%)				< 0.001	39.404
Moderate current drinking	5940 (97.9)	4967 (98.5)	973 (95.4)		
Excessive current drinking	125 (2.1)	78 (1.5)	47 (4.6)		
Smoking status, n (%)				0.83	0.372
Current smoking	1156 (20.6)	961 (20.7)	195 (20.1)		
Former smoking	1322 (23.6)	1087 (23.5)	235 (24.3)		
Never smoking	3124 (55.8)	2586 (55.8)	538 (55.6)		

Table 4 describes the relationship between biochemical indicators of depressive disorders in men. In the crude model, alanineamino transferase (ALT), alkaline phosphatase (AKP), bicarbonate, and chloride were all significantly linked to depression, yet neither model 1 nor model 2 exhibited these same associations. Albumin and total bilirubin (Tbil) levels were lower in the depressed group than in the control group in all models (*p* <  0.05). Gamma-glutamyl transferase (GGT), glucose, and triglycerides levels were higher in the depressed group than in the control group in all models (*p* <  0.05).

**Table 4:** Relationship between depressive disorders and biochemical indicators in men

Variable			*Crude model*		*Model 1*		*Model 2*	
	Crude OR (95% CI)	*P*	Adjusted OR (95% CI)^a^	*P*^*a*^	Adjusted OR (95% CI)^b^	*P*^*b*^	Adjusted OR (95% CI)^c^	*P*^*c*^
**Albumin (g/L)**	**0.94 (0.92 ~ 0.96)**	**< 0.001**	**0.95 (0.93 ~ 0.97)**	**< 0.001**	**0.95 (0.93 ~ 0.98)**	**0.001**	**0.96 (0.93 ~ 0.99)**	**0.013**
Alanineamino transferase (U/L)	1 (1 ~ 1)	0.112	1 (1 ~ 1)	0.044	1 (1 ~ 1.01)	0.033	1 (1 ~ 1.01)	0.166
Aspartate aminotransferase (U/L)	1 (1 ~ 1.01)	0.002	1 (1 ~ 1.01)	0.003	1 (1 ~ 1.01)	0.272	1 (1 ~ 1.01)	0.285
Alkaline phosphatase (U/L)	1 (1 ~ 1)	0.111	1 (1 ~ 1)	0.087	1 (1 ~ 1.01)	0.161	1 (1 ~ 1.01)	0.228
Blood urea nitrogen (mmol/L)	1 (0.97 ~ 1.03)	0.851	0.97 (0.94 ~ 1.01)	0.129	1 (1 ~ 1)	0.087	0.96 (0.91 ~ 1.01)	0.124
Total calcium (mmol/L)	0.72 (0.34 ~ 1.52)	0.388	0.88 (0.41 ~ 1.91)	0.75	0.96 (0.91 ~ 1.01)	0.24	0.73 (0.25 ~ 2.14)	0.566
Cholesterol (mmol/L)	1.04 (0.98 ~ 1.11)	0.154	1.05 (0.99 ~ 1.11)	0.119	0.53 (0.19 ~ 1.52)	0.408	1.02 (0.94 ~ 1.1)	0.65
Bicarbonate (mmol/L)	0.97 (0.94 ~ 1)	0.049	0.97 (0.94 ~ 1)	0.043	1.03 (0.96 ~ 1.12)	0.482	0.99 (0.95 ~ 1.03)	0.747
Creatinine (umol/L)	1 (1 ~ 1)	0.011	1 (1 ~ 1)	0.082	0.99 (0.95 ~ 1.03)	0.457	1 (1 ~ 1)	0.267
**Gamma-glutamyl transferase (U/L)**	**1 (1 ~ 1)**	**< 0.001**	**1 (1 ~ 1)**	**< 0.001**	**1 (1 ~ 1)**	**0.013**	**1 (1 ~ 1)**	**0.016**
**Glucose (mmol/L)**	**1.06 (1.04 ~ 1.09)**	**< 0.001**	**1.06 (1.03 ~ 1.08)**	**< 0.001**	**1.06 (1.02 ~ 1.09)**	**0.002**	**1.05 (1.01 ~ 1.08)**	**0.012**
Iron (umol/L)	0.99 (0.98 ~ 1)	0.02	0.99 (0.98 ~ 1)	0.071	0.99 (0.97 ~ 1)	0.083	0.99 (0.98 ~ 1.01)	0.29
Lactate dehydrogenase (U/L)	1 (1 ~ 1)	0.059	1 (1 ~ 1)	0.135	1 (1 ~ 1)	0.19	1 (1 ~ 1)	0.538
Phosphorus (mmol/L)	0.89 (0.63 ~ 1.25)	0.498	1 (0.7 ~ 1.42)	0.992	1.17 (0.74 ~ 1.86)	0.496	1.28 (0.8 ~ 2.06)	0.298
**Total bilirubin (umol/L)**	**0.97 (0.96 ~ 0.98)**	**< 0.001**	**0.97 (0.96 ~ 0.98)**	**< 0.001**	**0.97 (0.95 ~ 0.99)**	**< 0.001**	**0.97 (0.95 ~ 0.99)**	**0.001**
Total protein (g/L)	0.99 (0.98 ~ 1.01)	0.359	1 (0.98 ~ 1.01)	0.888	0.99 (0.97 ~ 1.01)	0.359	0.99 (0.97 ~ 1.01)	0.39
**Triglycerides (mmol/L)**	**1.06 (1.02 ~ 1.09)**	**0.001**	**1.06 (1.02 ~ 1.1)**	**0.001**	**1.08 (1.03 ~ 1.12)**	**0.001**	**1.06 (1.01 ~ 1.11)**	**0.01**
Uric acid (umol/L)	1 (1 ~ 1)	0.694	1 (1 ~ 1)	0.73	1 (1 ~ 1)	0.479	1 (1 ~ 1)	0.055
Sodium (mmol/L)	0.98 (0.95 ~ 1.01)	0.167	0.98 (0.96 ~ 1.01)	0.193	1.01 (0.97 ~ 1.05)	0.661	1.01 (0.97 ~ 1.05)	0.641
Potassium (mmol/L)	1.28 (1.07 ~ 1.55)	0.008	1.21 (1 ~ 1.46)	0.052	1.17 (0.9 ~ 1.51)	0.243	1.2 (0.92 ~ 1.56)	0.185
Chloride (mmol/L)	0.98 (0.95 ~ 1)	0.02	0.98 (0.95 ~ 1)	0.019	1 (0.97 ~ 1.03)	0.849	1 (0.97 ~ 1.03)	0.763
Osmolality (mmol/Kg)	1.01 (1 ~ 1.02)	0.166	1 (0.99 ~ 1.02)	0.587	1.01 (1 ~ 1.03)	0.158	1.01 (0.99 ~ 1.03)	0.201
Globulin (g/L)	1.03 (1.01 ~ 1.04)	< 0.001	1.03 (1.01 ~ 1.04)	< 0.001	1.01 (0.99 ~ 1.03)	0.168	1.01 (0.99 ~ 1.03)	0.462

*Note*: *SE* standard error, *OR* odds ratio, *CI* confidence interval

^a^Adjusted for age, race

^b^Adjusted for age, race, education, ratio of family income to poverty, marital status, whether to drink too much alcohol

^c^Adjusted for age, race, education, ratio of family income to poverty, marital status, whether to drink too much alcohol, body mass index, waist circumference

Table 5 shows the relationship between biochemical indicators of depression in women. Female models 1, and 2 were adjusted for all confounding factors in male models 1, and 2 plus alcohol consumption status. ALT, AKP, aspartate aminotransferase (AST), creatinine, GGT, glucose, iron, lactate dehydrogenase (LDH), Tbil, triglycerides, and globulin were significantly associated with depression in the crude model, but none of these indicators were significantly associated with depression in both model 1 and model 2. Although glucose and depression were strongly linked in models 1 and 2, sodium and depression were not connected even in the crude model. In contrast to the crude model and model 1, model 2 discovered a strong link between bicarbonate levels and depression. ALP, bicarbonate, and sodium levels were higher in the depressed group than in the control group in all models (*p* <  0.05).

**Table 5:** Relationship between depressive disorders and biochemical indicators in women

Variable			*Crude model*		*Model 1*		*Model 2*	
	Crude OR (95% CI)	*P*	Adjusted OR (95% CI)^a^	*P*^*a*^	Adjusted OR (95% CI)^b^	*P*^*b*^	Adjusted OR (95% CI)^c^	*P*^*c*^
Albumin (g/L)	1.01 (1 ~ 1.01)	0.001	1.01 (1 ~ 1.01)	0.001	0.98 (0.94 ~ 1.02)	0.33	1 (0.95 ~ 1.04)	0.834
Alanineamino transferase (U/L)	1.01 (1 ~ 1.01)	0.001	1 (1 ~ 1.01)	0.002	1.01 (1 ~ 1.01)	0.15	1.01 (1 ~ 1.01)	0.213
Aspartate aminotransferase (U/L)	1.01 (1 ~ 1.01)	0.001	1.01 (1 ~ 1.01)	0.001	1.01 (1 ~ 1.02)	0.074	1.01 (1 ~ 1.02)	0.057
**Alkaline phosphatase (U/L)**	**1.01 (1 ~ 1.01)**	**< 0.001**	**1.01 (1 ~ 1.01)**	**< 0.001**	**1.01 (1 ~ 1.01)**	**0.02**	**1.01 (1 ~ 1.01)**	**0.032**
Blood urea nitrogen (mmol/L)	0.99 (0.97 ~ 1.02)	0.515	0.98 (0.95 ~ 1.01)	0.122	0.99 (0.92 ~ 1.06)	0.703	0.99 (0.92 ~ 1.07)	0.807
Total calcium (mmol/L)	0.81 (0.46 ~ 1.44)	0.48	0.78 (0.44 ~ 1.38)	0.392	0.58 (0.13 ~ 2.53)	0.467	0.68 (0.15 ~ 3.14)	0.622
Cholesterol (mmol/L)	1.04 (0.99 ~ 1.09)	0.128	1.04 (0.98 ~ 1.09)	0.185	1.06 (0.93 ~ 1.21)	0.397	1.07 (0.94 ~ 1.23)	0.317
**Bicarbonate (mmol/L)**	**0.99 (0.97 ~ 1.02)**	**0.508**	**0.99 (0.96 ~ 1.01)**	**0.218**	**1.05 (0.99 ~ 1.11)**	**0.092**	**1.06 (1 ~ 1.13)**	**0.042**
Creatinine (umol/L)	1 (1 ~ 1)	< 0.001	1 (1 ~ 1)	< 0.001	1 (0.99 ~ 1.01)	0.937	1 (1 ~ 1.01)	0.756
Gamma-glutamyl transferase (U/L)	1 (1 ~ 1.01)	< 0.001	1 (1 ~ 1)	< 0.001	1 (1 ~ 1.01)	0.217	1 (1 ~ 1.01)	0.443
Glucose (mmol/L)	1.07 (1.04 ~ 1.09)	< 0.001	1.06 (1.04 ~ 1.09)	< 0.001	0.96 (0.89 ~ 1.04)	0.36	0.94 (0.87 ~ 1.03)	0.182
Iron (umol/L)	0.99 (0.98 ~ 1)	0.019	0.99 (0.98 ~ 1)	0.046	0.99 (0.97 ~ 1.01)	0.496	0.99 (0.97 ~ 1.02)	0.659
Lactate dehydrogenase (U/L)	1 (1 ~ 1)	0.003	1 (1 ~ 1)	0.01	1 (1 ~ 1.01)	0.239	1 (1 ~ 1.01)	0.287
Phosphorus (mmol/L)	1.18 (0.87 ~ 1.59)	0.29	1.22 (0.9 ~ 1.64)	0.206	1.69 (0.77 ~ 3.7)	0.188	1.82 (0.82 ~ 4.06)	0.141
Total bilirubin (umol/L)	0.99 (0.97 ~ 1)	0.021	0.99 (0.97 ~ 1)	0.024	0.99 (0.95 ~ 1.03)	0.533	1 (0.96 ~ 1.03)	0.887
Total protein (g/L)	1 (0.99 ~ 1.01)	0.934	1 (0.99 ~ 1.01)	0.934	0.98 (0.95 ~ 1.01)	0.195	0.99 (0.95 ~ 1.02)	0.374
Triglycerides (mmol/L)	1.14 (1.09 ~ 1.19)	< 0.001	1.14 (1.09 ~ 1.2)	< 0.001	1.11 (0.97 ~ 1.28)	0.135	1.07 (0.92 ~ 1.25)	0.358
Uric acid (umol/L)	1 (1 ~ 1)	0.037	1 (1 ~ 1)	0.053	1 (1 ~ 1)	0.639	1 (1 ~ 1)	0.286
**Sodium (mmol/L)**	**1 (0.98 ~ 1.03)**	**0.73**	**1 (0.98 ~ 1.02)**	**0.958**	**1.07 (1.01 ~ 1.12)**	**0.02**	**1.08 (1.02 ~ 1.14)**	**0.01**
Potassium (mmol/L)	1.04 (0.9 ~ 1.22)	0.575	1.03 (0.88 ~ 1.2)	0.734	1.42 (0.95 ~ 2.1)	0.084	1.36 (0.9 ~ 2.04)	0.141
Chloride (mmol/L)	0.99 (0.97 ~ 1)	0.163	0.99 (0.97 ~ 1)	0.154	1.02 (0.98 ~ 1.07)	0.307	1.03 (0.98 ~ 1.09)	0.183
Osmolality (mmol/Kg)	1.01 (1 ~ 1.02)	0.01	1.01 (1 ~ 1.02)	0.051	1.02 (1 ~ 1.05)	0.094	1.03 (1 ~ 1.05)	0.072
Globulin (g/L)	1.02 (1.01 ~ 1.03)	0.002	1.02 (1.01 ~ 1.03)	0.005	0.99 (0.96 ~ 1.02)	0.549	0.99 (0.95 ~ 1.02)	0.428

*Note*: *SE* standard error, *OR* odds ratio, *CI* confidence interval

^a^Adjusted for age, race

^b^Adjusted for age, race, education, ratio of family income to poverty, marital status, alcohol intake status, whether to drink too much alcohol

^c^Adjusted for age, race, education, ratio of family income to poverty, marital status, alcohol intake status, whether to drink too much alcohol, body mass index, waist circumference

**Fig. 2:**
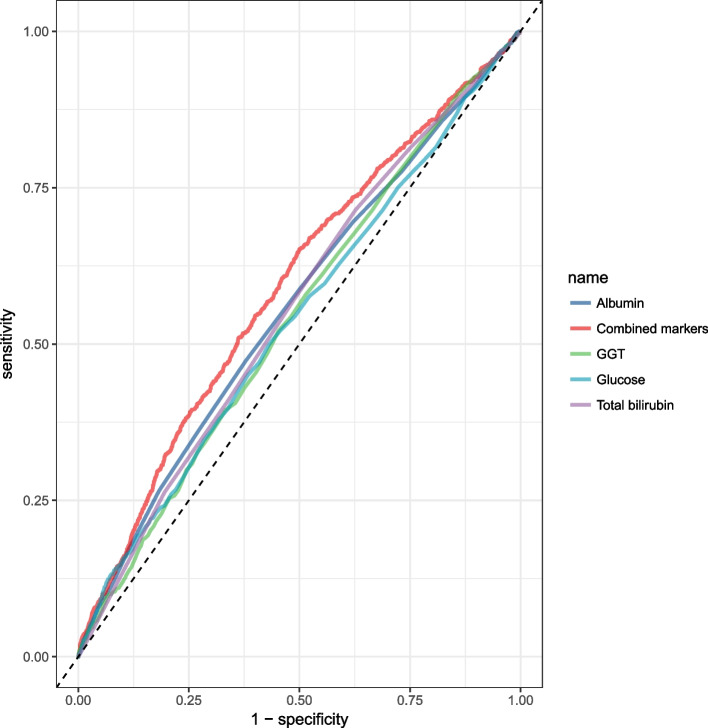
ROC curves for all single and combined markers in men

**Fig. 3:**
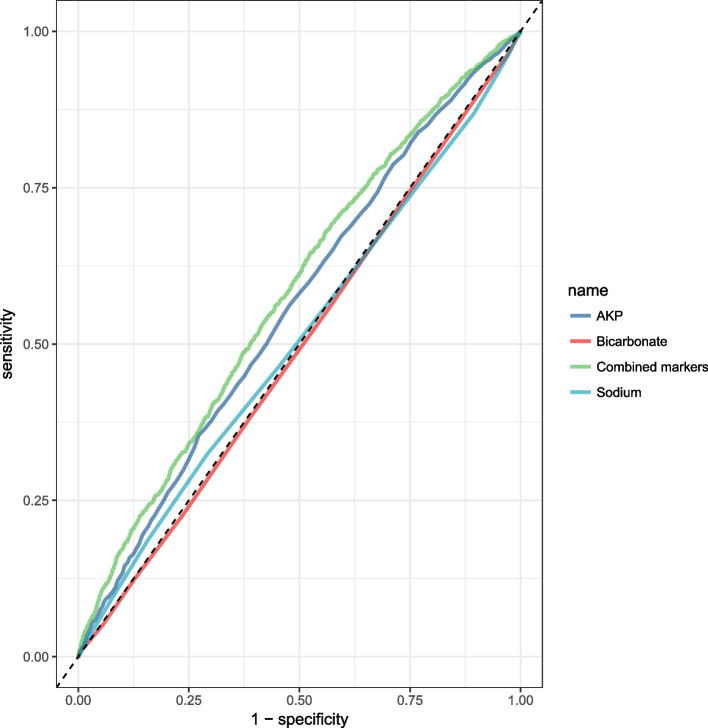
ROC curves for all single and combined markers in women

The biochemical indicator variables were included in stepwise logistic regression analysis and the regression coefficients of these biochemical indicators were used to calculate logit equations for assessing depressed male and female patients. In male patients, the logarithm of odds = − 0.112 − 0.042 (Albumin) + 0.002 (GGT) + 0.046(Glucose) − 0.027 (Tbil) (Table 6). In female patients, the logarithm of odds = − 1.629 -0.018 (Albumin) + 0.002 (GGT) + 0.033(Glucose) + 0.003 (AKP) + 0.002(Creatinine) + 0.125(Triglycerides) (Table 7). Based on the results of the Hosmer-Lemeshow test (*p* = 0.059, Chi-square = 15.032), this computational model was evaluated in women. But this computational model wasn’t evaluated in men (*p* = 0.017, Chi-square = 18.631). The sensitivity, specificity, and area under the curve (AUC) of these biomarker combinations were calculated separately in depressed patients (Tables 8 and 9; Figs. 2 and 3). The combined AUC was 0.592 (95% CI: 0.57-0.61) in men, indicating that they were more effective than all single markers in identifying depressed patients.

**Table 6:** Logistic regression analysis of variables associated with depression in men

Variable	B	SE	Wald	df	***P***	OR	95% C.I.
Albumin (g/L)	-0.042	0.010	18.080	1	0.000	0.959	0.941-0.978
GGT (U/L)	0.002	0.001	11.165	1	0.001	1.002	1.001-1.003
Glucose (mmol/L)	0.046	0.012	14.561	1	0.000	1.047	1.023-1.072
Tbil (μmol/L)	-0.027	0.007	16.009	1	0.000	0.973	0.960-0.986
Constant	−0.112	0.435	0.066	1	0.797	0.894	

*Note*: *B* partial regression coefficient, *SE* standard error, *df* degree of freedom, *OR* odds ratio, *CI* confidence interval

**Table 7:** Logistic regression analysis of variables associated with depression in women

Variable	B	SE	Wald	df	*P*	OR	95% C.I.
Albumin (g/L)	−0.018	0.008	4.846	1	0.028	0.982	0.967-0.998
GGT (U/L)	0.002	0.001	6.937	1	0.008	1.002	1.000-1.003
Glucose (mmol/L)	0.033	0.012	7.947	1	0.005	1.034	1.010-1.058
AKP (U/L)	0.003	0.001	8.553	1	0.003	1.003	1.001-1.005
Creatinine (μmol/L)	0.002	0.001	9.752	1	0.002	1.002	1.001-1.004
Triglycerides (mmol/L)	0.125	0.025	24.452	1	0.000	1.133	1.078-1.190
Constant	−1.629	0.372	19.206	1	0.000	0.196	

*Note*: *B* partial regression coefficient, *SE* standard error, *df* degree of freedom, *OR* odds ratio, *CI* confidence interval

**Table 8:** Estimated performances of all single markers and combined markers by ROC curve in men

Variable	Sensitivity	Specificity	AUC	95%CI
Albumin (g/L)	0.473	0.621	55.99	0.54-0.58
GGT (U/L)	0.522	0.547	54.33	0.52-0.56
Glucose (mmol/L)	0.499	0.569	53.83	0.52-0.56
Tbil (μmol/L)	0.715	0.373	55.67	0.54-0.58
Combined markers	0.652	0.500	59.16	0.57-0.61

**Table 9:** Estimated performances of all single markers and combined markers by ROC curve in women

Variable	Sensitivity	Specificity	AUC	95%CI
AKP (U/L)	0.562	0.523	55.75	0.54-0.57
Bicarbonate (mmol/L)	0.001	0.996	49.36	0.48-0.51
Sodium (mmol/L)	0.324	0.709	50.52	0.49-0.52
Combined markers	0.646	0.476	58.19	0.57-0.60

## Conclusion and directions for future research

In this large cross-sectional study, men in the depressed group had significantly higher levels of GGT, glucose, and triglycerides. Depressed men had lower albumin and total bilirubin levels than control men. As compared to the control group, women with depression had higher levels of AKP, Bicarbonate, and Sodium. For combined markers in men, the area under the curve was around 59.16%. The area under the curve for combined markers in women was 58.19%.

There has been a conflicting relationship between biochemical indicators and depression found in many studies to date. To provide some strong support, some large cross-sectional studies are lacking.

In the liver, kidney, and pancreas, GGT is primarily observed. Currently, GGT is the most sensitive enzyme indicator for liver diseases and is used to diagnose and monitor hepatobiliary diseases. Huang et al. reported that GGT was higher in NAFLD patients with depression than in patients without depression [12]. A positive association was also found between GGT levels and depression in men.

Kahn reported that fasting blood glucose levels were higher in depressed patients and these levels were significantly associated with depression scores [13]. Our findings are consistent with this. Loss of appetite is a common feature of depression, which can adversely affect blood glucose levels. In depressed patients, this difference may be caused by defects in glucose metabolism in brain regions such as the amygdala, prefrontal cortex, and hippocampus [14, 15].

We found that higher triglycerides was associated with depressive symptoms, and higher triglycerides subjects had higher levels of depression than normal subjects [16]. Triglycerides levels were significantly higher in the depressed group of men. In addition to lowering cholesterol and increasing triglycerides, interleukin-2 inhibits melatonin release, which reduces brain serotonin, resulting in depression and suicidal tendencies [17].

According to Pascoe MC, a low serum albumin level after stroke was associated with long-term depression symptoms in elderly Swedish patients [18]. We also found that serum albumin was negatively associated with depression.

Peng YF found correlations between BUN, fasting blood-glucose (FBG), TBil, and MDD in a Chinese Han population [8]. Bilirubin is an endogenous antioxidant, and low blood bilirubin levels are associated with seasonal depression, according to Shcherbinina MB [19]. Our results are further confirmed by this.

Recently, sodium was shown to modulate oxidative stress and inflammation, alter the autonomic nervous system, and cause innate and adaptive immune dysfunction [20]. .Many studies have shown that high sodium and chloride are directly associated with depression [21]. Women in the depressed group in our study tended to have higher levels of Sodium, which is more consistent with previous studies.

A measure of bone production, bone-specific alkaline phosphatase, was shown by Cizza G to be significantly greater in women with MDD than in controls [22]. Tissue non-specific alkaline phosphatase (TNAP), a globally expressed enzyme, is known for its activity in bone mineralization. Vitamin B6 molecules are calcified and transportable when this enzyme metabolizes phosphate compounds. Hypophosphatemia (HPP) is an uncommon metabolic disorder caused by hereditary loss-of-function mutations in the ALPL gene. In addition to decreased mineralization of bones and teeth, this systemic illness is also associated with anxiety disorders, seizures, and depression [23].

The study has some limitations. First, although though this study had a high sample size, the study group was only composed of Americans, and our findings might not be applicable to other nations due to variations in socio-demographic traits. Our findings also do not suggest a cause-and-effect link because this study was cross-sectional in nature. Finally, health status at the time of blood collection may affect biomarker results, and therefore the effect of certain disease information not obtained during the survey on biomarkers cannot be excluded. Despite these limitations of our results, the statistical results and findings in this study are robust due to the large-scale data.

In this study, a large sample size was analyzed, and a combined marker was constructed for both males and females, and the combined marker had a higher diagnostic value compared to the individual markers. Although similar studies have been conducted previously, the sample size was small, or the data collection was incomplete. This study provides a comprehensive analysis of 17,561 depressed patients from the NHANES database and provides some insight into the lack of laboratory indicators for depression diagnosis.

## Data Availability

The datasets generated and analyzed during the current study are available on the NHANES website: https://www.cdc.gov/nchs/nhanes/index.htm
